# Epidemiology and associated risk factors for injuries in collegiate cheerleading: a systematic review

**DOI:** 10.3389/fspor.2026.1714122

**Published:** 2026-06-17

**Authors:** Joylynn L. T. Haymes, Jeffrey S. Brooks, Amanda M. Black

**Affiliations:** 1 Department of Kinesiology, Faculty of Applied Health Sciences, Brock University, St Catharines, ON, Canada; 2 Department of Community Health Sciences, Cumming School of Medicine, University of Calgary, Calgary, AB, Canada

**Keywords:** athletic injuries, brain concussion, cheerleading, collegiate, female, sports medicine, sprains and strains

## Abstract

**Context:**

Collegiate cheerleading is a physically demanding sport with high rates of injuries. However, existing injury surveillance is limited, outdated, and inconsistent, underscoring the need to synthesize available evidence to guide prevention strategies.

**Objective:**

To investigate the epidemiology and risk factors for injuries in collegiate cheerleading by synthesizing evidence on injury rates, mechanisms, and types.

**Data sources:**

Eight electronic databases (PubMed, Embase, Web of Science CORE, Web of Science COMPLETE, Science Direct, OMNI, SPORTDiscus, MEDLINE, and CINAHL) were searched from inception to 19 October 2024.

**Study selection:**

Eligible studies were peer-reviewed, published in English from 2004 onward, and reported injury rates or risk factors in collegiate cheerleaders aged ≥ 18 years. Review articles and studies with ≤2 participants were excluded.

**Study design:**

Systematic review.

**Level of evidence:**

Level 3.

**Data extraction:**

Risk of bias and level of evidence were assessed using the Scottish Intercollegiate Guidelines Network validity scoring system and Oxford Centre for Evidence-Based Medicine criteria. Findings were narratively synthesized.

**Results:**

Six studies met inclusion criteria: one retrospective study (*n* = 184 female collegiate cheerleaders), four studies from a shared cohort of 9,022 athletes (96% female), and one case series of 54 catastrophic injuries. Injury rates ranged from 2.4 to 2.8 per 1,000 athlete-exposures. Sprains/strains, concussions, and fractures were the most common injury types. Stunting activities and practice sessions were the most frequent contexts for injury, with catastrophic injuries predominantly involving the head and neck. Inconsistent injury definitions and underreporting limited comparability.

**Conclusions:**

The evidence base is limited by outdated data, descriptive observational designs, self-reported injuries, and inconsistent definitions, restricting generalizability. Collegiate cheerleading presents substantial musculoskeletal injury risk, particularly during stunting and practice. Standardized injury surveillance, targeted prevention strategies, and high-quality, prospective research are needed to identify risk factors and evaluate interventions.

## 1 Introduction

Cheerleading remains a popular activity in the United States, with participation among individuals aged six years and older averaging about 3.5 million annually (1). At the collegiate level, routines include advanced tumbling, multi-level stunts, and elevated pyramids that involve performance at height and complex coordinated movements (2). These elements are associated with an elevated risk of catastrophic injury (3–7), defined by the National Center for Catastrophic Sports Injury Research (NCCSIR) as “any injury incurred during participation in a high school/college-sponsored sport, in which there is permanent severe functional neurological disability (nonfatal) or transient but not permanent functional neurological disability (serious)” (6). Incidence rates of such injuries are significantly higher at the collegiate level compared to high school (5, 7–9), with risks linked to factors such as landing surfaces, lack of safety training, improper technique, and inadequate supervision during stunts and routines (5, 7–9). These risks may be amplified in collegiate cheerleading, where advanced skill demands and greater competitive intensity increase the likelihood of injury (5, 7–9). Prior to the International Cheer Union's (ICU) founding in 2004, cheerleading lacked standardized international governance, and catastrophic injury rates at the collegiate level were notably higher, attributable in part to increasingly complex stunts performed without consistent safety regulations (4). The present review focuses on the post-2004 era to capture injury epidemiology under a standardized competitive and regulatory framework, thereby improving relevance to contemporary prevention efforts.

Quantifying injury incidence and identifying risk factors are foundational steps in developing targeted prevention strategies in collegiate cheerleading (10). Collegiate cheerleading was selected as the focus of this review because there is limited epidemiological data specific to this population. Differences in institutional medical and performance support resources compared to club or high school settings, as well as population characteristics, may also influence injury risk and prevention approaches (11). Given the technical elements of collegiate cheerleading, injury mechanisms may differ from other sports, underscoring the need for sport-specific epidemiological synthesis. Previous reviews of cheerleading injury etiology have used narrative approaches and included a broad range of ages and competition levels (12, 13). While informative for introducing cheerleading epidemiological patterns, a systematic review focused on the collegiate setting, with attention to methodological rigour, is needed to guide future prospective cohort studies in this high-risk population. Accordingly, the objective of this systematic review was to synthesize studies reporting injury incidence, injury characteristics, and associated risk factors among collegiate cheerleading athletes.

## 2 Methods

### 2.1 Review design and reporting standards

This review followed the 2020 Preferred Reporting Items for Systematic Reviews and Meta-Analyses (PRISMA) guidelines (14), guided by the PERSiST recommendations for implementing PRISMA in sport and exercise medicine (15). The inclusion and exclusion criteria, search strategy, and methods for data extraction and synthesis were defined *a priori* before the search was conducted. This review was not prospectively registered.

### 2.2 Eligibility criteria

The eligibility criteria were defined *a priori* according to the Population, Intervention (Exposure), Comparator, Outcomes, and Study Design (PICOS) framework.

#### 2.2.1 Inclusion criteria

Studies were eligible for inclusion if they involved collegiate or university-level cheerleaders aged 18 years or older (Population) and examined injuries occurring during participation in collegiate cheerleading activities (Exposure). Eligible studies were required to report injury-related outcomes, including injury incidence, injury rates, locations, mechanisms, severity, or other epidemiological characteristics relevant to collegiate cheerleading (Outcomes). Studies that included multiple competition levels (e.g., high school, club, or youth) were eligible only if data specific to the collegiate subgroup could be extracted separately. All study designs capable of reporting original injury data were considered, including randomized controlled trials, cohort studies, case-control studies, and cross-sectional studies with more than two participants (Study Design). Only peer-reviewed studies published in English from 2004 onward were included, corresponding with the founding of the International Cheer Union and the sport's formalization and regulation (16). The English language restriction was applied due to resource limitations related to translation and screening capacity.

#### 2.2.2 Exclusion criteria

Studies were excluded if they were animal studies, review articles, opinion pieces, conference abstracts, or non–peer-reviewed publications. Articles that did not report injury rates, risk factors, or other outcomes specific to collegiate cheerleading, focused on athletes younger than 18 years, or were not available in full text were also excluded.

### 2.3 Information source and search strategy

A comprehensive literature search was conducted across eight electronic databases: PubMed, Embase, Web of Science CORE, Web of Science COMPLETE, Science Direct, OMNI, SPORTDiscus, MEDLINE via OVID, and CINAHL. Searches were performed from database inception to 19 October 2024. The search strategy was guided by the PICO framework and included two sets of keywords. The first set captured the population of interest, including terms for collegiate cheerleaders (“cheer*”, “cheerlead*”, “college*”, “collegiate*”, “NCAA*”, “university*”). The second set addressed injury outcomes and risk factors, including terms such as “injur*”, “concussion*”, “strain*”, “sprain*”, “fractur*”, “tear*”, “risk factor*”, and “incidence rate*”. Keywords within each set were combined using the OR operator, and the two sets were combined using the AND operator. Searches were limited to peer-reviewed publications in English, and the results were filtered to include studies published from 2004 onward, in line with the formalization of collegiate cheerleading by the International Cheer Union (16). Reference lists of all included studies and relevant reviews were also manually screened to identify additional relevant articles.

### 2.4 Study selection

All identified records were imported into Covidence (Covidence systematic review software, Veritas Health Innovation, Melbourne, Australia, www. covidence.org), which was used to remove duplicates and screen articles. The database search was conducted by the lead authors (JH & JB) under the guidance of the senior author (AB), who has extensive experience in systematic reviews. Titles and abstracts were independently screened for potential eligibility by the lead authors (JH & JB). Articles deemed potentially relevant were retrieved in full text and independently assessed for inclusion by the same two authors. Any discrepancies at either the title/abstract or full-text screening stages were first resolved through discussion between the two reviewers. If consensus could not be reached, a third reviewer (AB) was consulted to resolve disagreements.

### 2.5 Data collection process and data items

#### 2.5.1 Main outcome variables

The main outcome variables extracted from each included study were related to injury epidemiology and risk factors in collegiate cheerleaders. These included injury type, location, mechanism, severity, number of injuries, days missed due to injury, and whether injuries occurred during practice or competition. For studies examining interventions or risk factors, exposure status and any associated risk factors were recorded. Additional contextual variables included coaching certification or experience and athlete years of experience. These outcomes were selected to capture both the frequency and characteristics of injuries and to identify potential predictors or risk factors relevant to collegiate cheerleading.

#### 2.5.2 Data extraction

Data were independently extracted by two authors (JH and JB) using a standardized Excel extraction form. Following independent extraction, the authors compared their results and resolved any discrepancies through discussion and consensus. Extracted study-level data included study design, sample size, participant demographics (age range, sex distribution, level of cheerleading), classification of injury, and details of any interventions examined. Outcome measures and results recorded included injury location, mechanism, type, event, and incidence rate.

Given the absence of a cheerleading-specific extension to the International Olympic Committee (IOC) consensus statement for injury surveillance, terminology related to injury mechanism and activity at the time of injury varied across studies. Injury characteristics were extracted and reported according to the definitions and classifications used by the original authors. Where studies labeled variables as “mechanism”, these were presented as such, even when categories reflected the activity, role, or maneuver performed at the time of injury. No attempt was made to retrospectively reclassify mechanisms according to IOC contact-based categories in order to avoid reinterpretation of the original data.

Key findings, study implications, and limitations were also captured. No data were extracted from figures, and no assumptions were made about missing or unclear information.

### 2.6 Study risk of bias

The methodological quality and risk of bias of the included studies were assessed using the Scottish Intercollegiate Guidelines Network's (SIGN) validity checklist (17). The SIGN tool was selected because it provides study design-specific appraisal criteria for observational research and evaluates domains known to influence risk of bias, including participant selection, exposure and outcome measurement, and statistical analysis. Its structured approach allows for consistent appraisal across heterogeneous observational study designs. Population-based injury surveillance studies were evaluated using the SIGN checklist for cohort studies. The level of evidence for each study was determined using the Oxford Centre for Evidence-Based Medicine (OCEBM) framework (18), which provides a standardized hierarchy of evidence to contextualize study findings. Two authors (JH & JB) independently assessed each study for risk of bias and study quality. Discrepancies were resolved through discussion and consensus. No automation tools were used, and no contact with study authors was required to clarify study methods or results. It is noted that the SIGN tool and the OCEBM framework serve distinct and complementary purposes: SIGN evaluates methodological quality and risk of bias within a given study design, while OCEBM assigns a level of evidence based on study design hierarchy. A study may therefore receive an acceptable SIGN rating while being assigned a lower OCEBM level of evidence, reflecting the design-based ceiling on inferential strength rather than a contradiction between the two assessments.

### 2.7 Data synthesis and analysis

Due to the heterogeneity of study designs, outcomes, and reporting methods, a descriptive narrative synthesis was conducted for all included studies. Where multiple included studies were derived from the same underlying surveillance cohort, findings were extracted and presented at the individual study level and were not statistically combined, to mitigate the risk of evidence inflation. Consistency in findings across studies sharing a common data source was interpreted as reflecting shared data origin rather than independent corroboration and is noted accordingly in the synthesis. Outcomes were reported as presented in the original studies without transformation or statistical pooling. Effect measures were summarized using the metrics reported in each study, such as injury rates, injury types, locations, mechanisms, practice details, and participant or coach characteristics. All studies meeting the eligibility criteria were included in the synthesis. Data were presented in tabular format, organized to display study design, inclusion and exclusion criteria, injury classification, risk factors examined, outcome measures, results, limitations, and study quality. No quantitative meta-analysis, subgroup, or sensitivity analyses were conducted due to the heterogeneity and nature of the included studies.

## 3 Results

**Figure 1:**
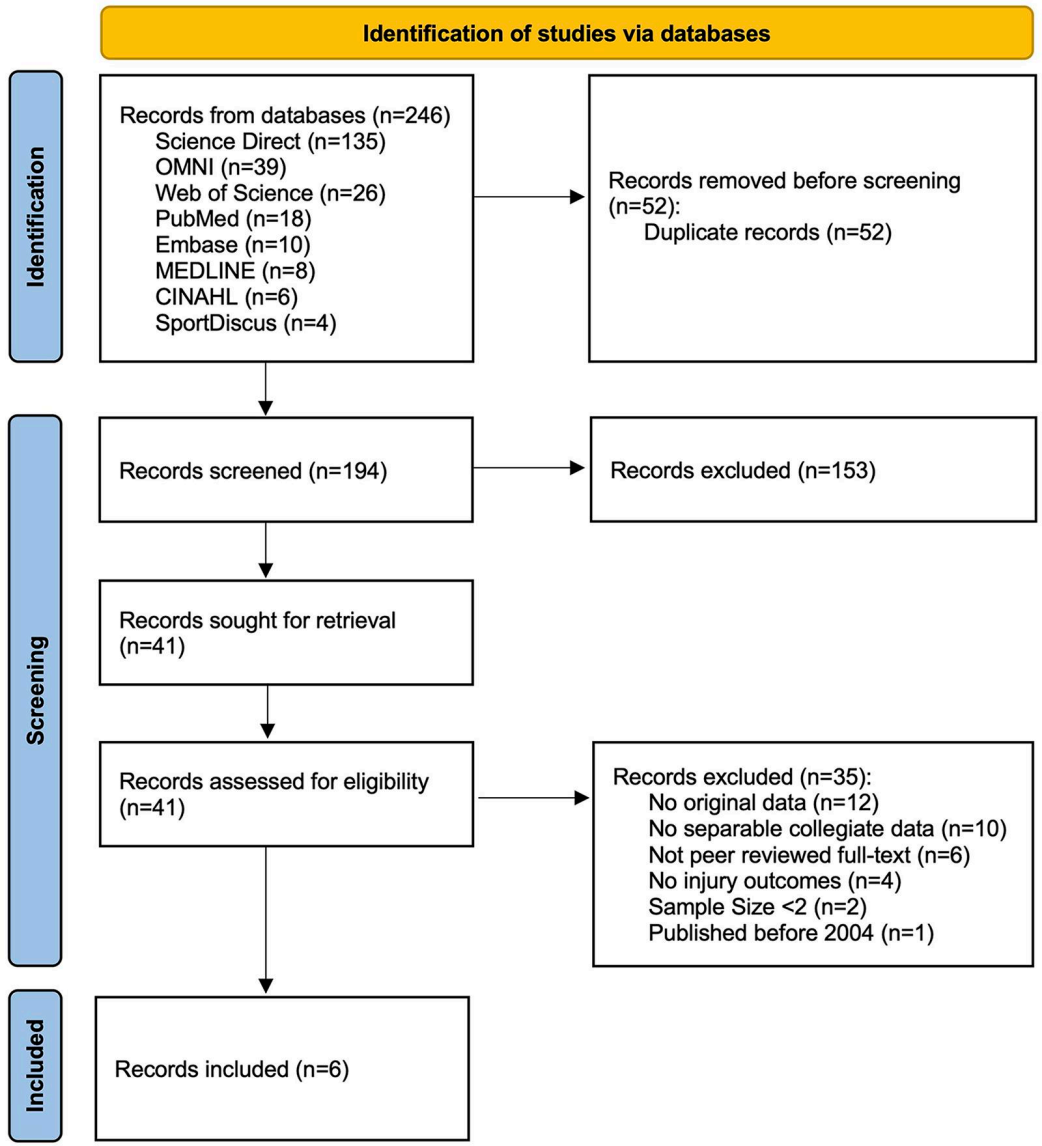
Preferred reporting items for systematic reviews and meta-analyses (PRISMA) flow chart of included and excluded studies for collegiate cheerleading injury literature.

The initial search identified 246 records (Figure 1). After duplicates were removed, 194 studies remained for title and abstract screening. Of these, 41 were selected for full-text review, resulting in six studies that met the inclusion criteria. Four of these studies drew from the same cohort, while the others were independent. These four publications were derived from the same underlying prospective surveillance cohort and analyzed overlapping years, each focusing on different injury characteristics (e.g., overall injuries, sprains/strains, stunt-related injuries, fall-related injuries). Findings were extracted and presented at the individual study level and were not statistically combined to avoid duplication or inflation of evidence. Similar results across these studies therefore reflect analyses of the same cohort rather than independent samples. During title and abstract screening, inter-rater agreement prior to consensus was 83.5%, with a Cohen's kappa of 0.53, indicating moderate agreement between reviewers (30).

### 3.1 Characteristics of all included studies

The included studies were published between 2005 and 2019. All but one relied on data collected before 2007, which examined catastrophic injury data only between 2002 and 2017 (19). One retrospective surveillance study (20), four prospective surveillance studies (5, 7–9), and one case series (19) were identified (Table 1). All studies were conducted in the United States and involved collegiate cheerleaders, although five also included high school, middle school, and elementary school athletes (5, 7–9, 19).

**Table 1:** Summary of included studies reporting epidemiology and risk factors for collegiate cheerleading injuries.

Authors and publication year	Study design	Data collection method	Setting /population	Incidence rate per 1,000 AE	Injury mechanism (as reported by authors)	Activity/maneuver at time of injury (as reported by authors)	Type of event	Type of injury	Location of injury
Jacobson et al. (20)	Retrospective Injury Surveillance	Self-reported questionnaire	23 US Division I universities; 184 female collegiate cheerleaders (mean age 20.2 ± 1.8, range 18–23)	2.8	Not reported	Stunt, *n* = 94 (51%)	Practice, *n* = 163 (88.8%) **Performance:** Game performance or competition, *n* = 21 (11.2%)	Not reported	Ankle, *n* = 83 (44.9%) Wrist/Hand, *n* = 35 (19.3%) Knee, *n* = 22 (11.9%) Head/Neck, *n* = 19 (10.2%) Back, *n* = 17 (9.2%) Thigh, *n* = 5 (2.7%) Face, *n* = 3 (1.8%)
Shields and Smith (8) Fall-related	Prospective Injury Surveillance	Internet-based surveillance system (Cheerleading RIO) with weekly reports submitted by trained team reporters	37 US college cheerleading teams (2006–2007) 16 injuries	0.29 (95% CI: 14.9–43.1)	Injured landed on another cheerleader, *n* = 16 Another landed on injured cheerleader, *n* = 6 Other athlete as base, *n* = 4 Another landed on and pinned injured cheerleader, *n* = 2 Injured as base, *n* = 1 Simultaneous multi-athlete landing, *n* = 1 Unknown, *n* = 2 *pooled across All-Star, college, and high school levels	Cradle, *n* = 1 (6.2%) Extension, *n* = 3 (18.8%) Miscellaneous stunt, *n* = 4 (25%) Pyramid, *n* = 3 (18.8%) Single-based stunt, *n* = 1 (6.2%) Single-leg stunt, *n* = 1 (6.2%) Stunt-cradle combo, *n* = 1 (6.3%) Transition, *n* = 2 (12.5%)	Practice, *n* = 12 (75.0%) **Performance:** Pep Rally, *n* = 1 (6.2%) Athletic Event, *n* = 2 (12.5%) Competition, *n* = 1 (6.3%)	Concussion, *n* = 4 (25.0%) Dislocation, *n* = 4 (25.0%) Soft tissue, *n* = 3 (18.8%) Strain/sprain, *n* = 3 (18.8%) ACL tear, *n* = 1 (6.3%) Other, *n* = 1 (6.2%)	Head/face, *n* = 4, (25.0%) Neck, *n* = 1 (6.3%) Upper Extremity, *n* = 2 (12.5%) Lower Extremity, *n* = 4 (25.0%) Trunk, *n* = 5 (31.3%)
Shields and Smith (5) Prospective Surveillance	Prospective Injury Surveillance	Internet-based surveillance system (Cheerleading RIO) with weekly reports submitted by trained team reporters	37 US college cheerleading teams (2006–2007) 133 injuries	Overall: 2.4 (95% CI: 2.0–2.8) Performance: 1.2 (95% CI: 0.7–1.7) Practice: 0.8 (95% CI: 0.7–0.9)	Basing/spotting, *n* = 37 (28.0%) Collided with 1 + cheerleaders, *n* = 11 (8.3%) Failed to complete maneuver, *n* = 25 (19.0%) Fell, *n* = 16 (12.1%) Slipped/tripped/twisted body part, *n* = 9 (6.8%) Jumping, *n* = 5 (3.8%) Tumbling, *n* = 23 (17.4%) Other, *n* = 6 (4.6%) Unknown, *n* = 1 (-)	Standing Tumbling, *n* = 11 (8.3%) Running Tumbling, *n* = 19 (14.3%) Jump, *n* = 4 (3.0%) General Stunt, *n* = 67 (50.4%) Dancing, *n* = 1 (0.7%) Other, *n* = 6 (4.5%)	Practice, *n* = 112 (84.2%) **Performance:** Pep rally, *n* = 3 (2.2%) Athletic event, *n* = 15 (11.3%) Competition, *n* = 3 (2.3%)	Strain/Sprain, *n* = 67 (50.4%) Soft tissue, *n* = 19 (14.3%) Fracture/dislocation, *n* = 16 (12.0%) Concussion, *n* = 12, (9.0%) Cartilage/ligament/tendon tear, *n* = 7 (5.3%) Other, *n* = 12 (9.0%)	Head/face, *n* = 25 (18.8%) Neck, *n* = 8 (6.0%) Upper Extremity, *n* = 27 (20.3%) Lower Extremity, *n* = 40 (30.1%) Trunk, *n* = 33 (24.8%)
Shields and Smith (9) Strain/sprain	Prospective Injury Surveillance	Internet-based surveillance system (Cheerleading RIO) with weekly reports submitted by trained team reporters	37 US college cheerleading teams (2006–2007) 65 injuries, 41 females mean age 20.1 ± 2.5, range 18–29	Overall: 1.2 (95% CI: 0.9–1.5) Practice: 1.3 Competition: 1.5 Athletic event: 0.7 Pep rally: 0.5	Contact with another cheerleader, *n* = 16 (5.7%) Failed to complete maneuver, *n* = 43 (15.4%) Fall, *n* = 40 (14.3%) Spotting/basing cheerleader, *n* = 53 (18.9%) Tumbling, *n* = 52 (18.6%) Twisted body part, *n* = 33 (11.8%) Other, *n* = 43 (15.4%) *pooled across All-Star, college, and high school levels	Tosses, *n* = 7 (2.5%) Standing tumbling, *n* = 42 (15.0%) Running tumbling, *n* = 49 (17.5%) Stunts, *n* = 129 (46.1%) Pyramid, *n* = 22 (7.9%) Jumps, *n* = 12 (4.3%) Dancing/other, *n* = 19 (6.8%) *pooled across All-Star, college, and high school levels	Not reported	Not reported	Neck, *n* = 6 (9.2%) Upper extremity, *n* = 14 (21.5%) Lower extremity, *n* = 22 (33.8%) Trunk, *n* = 23 (35.4%)
Shields and Smith (7) Stunt	Prospective Injury Surveillance	Internet-based surveillance system (Cheerleading RIO) with weekly reports submitted by trained team reporters	37 US college cheerleading teams (2006–2007) 88 injuries, 68 females mean age + SD = 19.6 ± 2.0	1.59	Catching, *n* = 14 (15.9%) Contact w/another cheerleader, *n* = 10 (11.4%) Failed to complete maneuver, *n* = 12 (13.6%) Fall, *n* = 16 (18.2%) Improper maneuver execution, *n* = 2 (2.3%) Lifting/tossing, *n* = 9 (10.2%) Spotting/basing, *n* = 6 (6.8%) Twisted body part, *n* = 3 (3.4%) Other, *n* = 16 (18.2%)	Cradle, *n* = 24 (27.3%) Extension, *n* = 5 (5.7%) Miscellaneous stunt, *n* = 17 (19.3%) Pyramid, *n* = 15 (17.0%) Single-based stunt, *n* = 8 (9.1%) Single-leg stunt, *n* = 8 (9.1%) Stunt-cradle combo, *n* = 5 (5.7%) Transition, *n* = 3 (3.4%) Unspecified partner stunt, *n* = 1 (1.1%) Unspecified group stunt, *n* = 2 (2.3%)	Practice, *n* = 76 (86.4%) **Performance:** Pep Rally, *n* = 2 (2.3%) Athletic Event, *n* = 8 (9.1%) Competition, *n* = 2 (2.2%)	Abrasion, contusion, or hematoma, *n* = 15 (17.0%) Concussion, *n* = 11 (12.5%) Fracture/dislocation, *n* = 9 (10.2%) Laceration/puncture, *n* = 3 (3.4%) Strain/sprain, *n* = 39 (44.3%) Other, *n* = 11 (12.6%)	Head, *n* = 12 (13.6%) Face, *n* = 11 (12.5%) Neck, *n* = 5 (5.7%) Trunk, *n* = 23 (26.1%) Upper extremity, *n* = 16 (18.2%) Lower extremity, *n* = 21 (23.9%)
Yau et al. (19)	Case Series	National surveillance system (NCCSIR) using reports from media, coaches, athletic trainers, sport organizations, and online portal.	National catastrophic injury surveillance; 13 traumatic catastrophic injuries in collegiate cheerleaders (2002–2017), 12 females	**Pre-rule change:** 1.75 per 1,000,000 cheerleaders (95%CI: 0.04–3.46) **Post-rule change:** 0.22 per 1,000,000 cheerleaders (95%CI: −0.21–0.64)	Not reported	Basket toss, *n* = 5, (38%) Pyramid, *n* = 2, (15%) Any other team formation involving 3 + persons, *n* = 2, (15%) Two-person stunt, *n* = 2 (15%) Tumbling and floor routine, *n* = 2 (15%)	Practice, *n* = 8 (62%) **Performance:** Competition, *n* = 5 (38%)	Fracture, *n* = 8 (62%) Traumatic brain injury, *n* = 3 (23%) Other, *n* = 1 (8%) Unknown, *n* = 1 (8%)	Brain/head, *n* = 6 (46%) Cervical spine, *n* = 6 (46%) Other spine, *n* = 1, (8%)

Injury mechanism and activity at time of injury were extracted and presented as reported by the original authors. Mechanism categories reflect event-based descriptions used by included studies and do not correspond to standardized contact-based classifications. Where studies reported only activity, role, or maneuver performed at the time of injury under a mechanism label, data were retained under the original authors’ terminology to avoid reinterpretation.

### 3.1.1 Participants and demographics

All studies primarily involved female athletes, but sex reporting was inconsistent. One study limited participants to females only (*n* = 184) (20). The shared cohort studies provided sex counts of injured athletes [20 male, 68 female (7); 24 male, 41 female (9)] in some but not all studies, and did not consistently report total athlete-exposures or team-level demographics (7, 9). Two of the shared cohort studies noted that 96% of participants were female but did not provide a complete sex breakdown or team counts (5, 8). The case series described 12 female and one male athlete injuries but estimated denominators for exposure rates using publicly available participation data (19).

### 3.1.2 Injury definition and incidence

Five studies (83%) defined an injury as one that required medical attention and resulted in at least one day of missed participation in cheerleading (5, 7–9, 20). The case series did not directly define injury but classified catastrophic injuries into severity categories (serious, nonfatal, fatal; 19).

Denominators also varied. The shared cohort studies reported incidence by athlete-exposures (5, 7–9), the case series reported per 1,000,000 cheerleaders (19), while the retrospective study provided only raw injury counts (20).

### 3.1.3 Injury type and location

Reporting of injury type was often incomplete. Only catastrophic injury categories (fracture, traumatic brain injury, other) were reported in one study (19), while another did not report injury type at all (20). All studies reported injury location, but specificity varied; for example, some grouped injuries only as “upper” or “lower” extremities (5, 7).

### 3.1.4 Injury mechanism and context

The four shared cohort studies reported injury mechanisms using event-based categories (e.g., collision with another cheerleader, fall, failed maneuver execution; 5, 7–9), while all six studies reported the activity, role, or maneuver being performed at the time of injury (e.g., tumbling, stunting, basing/spotting) (5, 7–9, 19, 20). However, two of the shared cohort studies did not stratify mechanism by competition level (8, 9). None of the included studies classified mechanisms according to IOC contact-based categories (direct contact, indirect contact, non-contact). Reporting of activity context, such as practice, competition, or other events, was inconsistent, with some studies distinguishing only practice vs. competition (19, 20).

### 3.1.5 Risk factors and prevention

Assessment of injury risk factors and preventive interventions was limited across included studies. One study evaluated a rule change after prohibiting basket tosses on hard surfaces (19). None of the included studies explicitly examined injury risk factors using analytical methods, nor investigated preventive strategies or implementation effectiveness. Reporting of medical and safety resource availability was limited across studies. Only one study reported team-level access to certified athletic trainers (ATs), indicating that approximately 52% of collegiate cheerleading programs had athletic training support.

## 3.2 Risk of bias assessment

Methodological quality and risk of bias were evaluated using the SIGN validity checklist (17). The one retrospective cohort study (20) was rated as “0” (unacceptable quality), based on several methodological concerns identified through the SIGN checklist, including reliance on a self-reported questionnaire with no validation of injury data, absence of a clearly defined exposure denominator, and lack of prospective outcome tracking. In contrast, the four prospective surveillance shared cohort studies (5, 7–9) were rated as “+” (acceptable quality), reflecting their use of a standardized internet-based surveillance system, predefined injury definitions, and systematic weekly exposure reporting, despite limitations including voluntary institutional participation and reliance on team personnel for data entry. The case series (19) was also rated as “+” (acceptable quality) given its use of a national registry with established data collection procedures, while recognizing inherent limitations of case series designs including absence of a comparison group and potential underascertainment (Table 2).

**Table 2:** Methodological quality and level of evidence of included studies reporting epidemiology and risk factors for collegiate cheerleading injuries based on the Scottish intercollegiate guidelines network (SIGN) validity checklist (17) and the Oxford centre for evidence-based medicine framework (18).

Authors and publication year	Scottish Intercollegiate Guidelines Network (SIGN) validity score	Oxford Centre for Evidence-Based Medicine Level of Evidence
Jacobson et al. (20)	0 (Unacceptable)	3
Shields and Smith (8) Fall-related	+ (Acceptable)	3
Shields and Smith (5) Prospective Surveillance	+ (Acceptable)	3
Shields and Smith (9) Strain/sprain	+ (Acceptable)	3
Shields and Smith (7) Stunt	+ (Acceptable)	3
Yau et al. (19)	+ (Acceptable)	4

SIGN scoring: ++ = high quality; + = acceptable quality; 0 = unacceptable quality.

Correspondingly, the level of evidence for the included studies was low to moderate based on the OCEBM framework (18). The five cohort studies were rated as Level 3 evidence (5, 7–9, 20), while the registry-derived case series was rated as Level 4 evidence (19; Table 2).

The cohort studies relied on voluntary or self-reported injury and exposure data entered by team personnel (5, 7–9, 20). Although surveillance procedures typically included predefined injury definitions, exposure tracking, and regular data audits, none of the studies employed population-based sampling strategies. The case series derived from the National Centre for Catastrophic Sport Injury Research was based on registry data of catastrophic events (19).

## 3.3 Epidemiology and patterns of injury

In surveillance-based studies, the majority of injuries in collegiate cheerleading occurred during practice [range: 83% (5, 7–9)–88.8% (20)], with stunts being the most frequent reported activity at the time of injury, accounting for approximately half of all injuries [51% (20) −52% (5, 7–9)]. Injury rates per 1,000 athlete-exposures were comparable across surveillance studies [2.4 (5)–2.8 (20)], and both reported similar injury location distributions. Ankle injuries were the most frequent non-catastrophic injury (44.9%) (20), followed by wrist and hand injuries (20), while sprains/strains (8, 9) and concussions (7, 8) were also common across studies. Catastrophic injuries primarily involved the head and neck, often resulting from falls and improper landings during stunts, particularly among flyers (19). Following the 2006–2007 rule change that prohibited basket tosses on hard surfaces, the frequency of catastrophic injuries decreased by 74% (Table 1; 19). Injury patterns also varied across activity type and cheerleading role (e.g., base vs. flyer). Access to a dedicated athletic trainer was reported by 45.9% (5) to 52.3% (7) of teams. Injury definitions and reliance on self-reported data varied across studies, contributing to heterogeneity in reported outcomes.

## 4 Discussion

The available literature on collegiate cheerleading injuries is limited in both quantity and methodological depth, with only six eligible studies identified, four of which analyzed overlapping surveillance datasets. Reported injury rates in participating collegiate cheerleading programs ranged from 2.4 (5) to 2.8 (20) per 1,000 athlete exposures, suggesting relatively lower observed injury rates compared with men's football (21), women's gymnastics (22), and women's soccer (23). However, interpretation of these estimates is constrained by substantial heterogeneity in study design, reporting methods, and outcome classification. Beyond reporting heterogeneity, a more fundamental limitation is the scarcity of contemporary longitudinal surveillance studies capable of supporting risk factor identification and prevention effectiveness evaluation.

Across included studies, injury surveillance practices were not fully aligned with international sport injury reporting standards established by the International Olympic Committee (24). Definitions of injury, anatomical coding schemes, mechanism classification, and contextual exposure measurement varied substantially. Most studies restricted surveillance to injuries requiring medical attention and time loss, while one focused primarily on catastrophic events. Reporting of athlete demographic characteristics was also inconsistent, limiting assessment of population representativeness. Collectively, these limitations reduce comparability across studies and restrict the utility of existing evidence for comprehensive injury burden estimation. The predominance of descriptive surveillance designs reflects an early stage of evidence development for collegiate cheerleading injury epidemiology. Advancing this literature will require prospective surveillance systems that incorporate standardized injury definitions, detailed mechanism coding, and contextual exposure tracking consistent with international guidance. Given these surveillance limitations, interpretation of injury patterns must be considered within the context of evolving prevention and safety strategies in collegiate cheerleading.

The high proportion of injuries sustained during stunts [51% (20)], combined with the frequency of falls and improper landings (19), highlights the elevated mechanical risk associated with high-elevation cheerleading maneuvers. Basket tosses, pyramids, and cradles are particularly hazardous when performed on hard surfaces (19). The 2006 rule change prohibiting basket tosses on hard surfaces resulted in a 74% reduction in catastrophic injuries (19), demonstrating the potential effectiveness of targeted regulatory interventions. Building on this precedent, environmental safety controls, including compliant performance surfaces, enhanced spotter and base training protocols, and sustained access to certified athletic trainers, are important considerations for reducing catastrophic injury risk.

Another strategy to reduce injury burden in cheerleading is the implementation of structured injury prevention programs (25). Future programs may benefit from prioritizing strength and conditioning interventions targeting lower extremity stability, given that ankle injuries accounted for 44.9% (20) of reported cheerleading injuries, though cheerleading-specific validation of such approaches is currently lacking. Evidence from a systematic review of balance training programs in soccer reported a pooled 36% reduction in ankle injury rates compared to standard warm-up routines (26). Evidence from adjacent sport populations suggests that neuromuscular training approaches may represent a plausible component of future cheerleading injury prevention programs, though the applicability of these findings to cheerleading is limited by the absence of cheerleading-specific trials. Randomized controlled trials evaluating the efficacy of such programs in collegiate cheerleading populations are needed before practice recommendations can be made with confidence (31).

Training optimization for athletes performing base and spotting roles represents another potential injury prevention strategy. Proper lifting biomechanics and spotting procedures may reduce acute injury risk during stunt execution, particularly for athletes supporting flyers during pyramids and basket tosses. Although no randomized controlled trials have evaluated cheerleading-specific prevention programs targeting these roles, observational implementation studies have reported improvements in performance safety and reductions in severe injury outcomes (4, 19).

Access to qualified medical personnel also appears to be an important structural factor in injury prevention. Only 52% of collegiate cheerleading teams reported access to athletic trainers (7). Athletic trainers provide rapid injury assessment, support concussion management, and improve documentation accuracy, thereby reducing reporting bias associated with coach- or athlete-entered surveillance systems (27). Teams with AT support have reported reductions in injuries such as ankle sprains and concussions, likely reflecting enhanced supervision and early clinical intervention (27).

Targeted educational initiatives for coaches and athletic staff may further contribute to injury risk reduction in collegiate cheerleading. Most injuries [83% (5, 7–9)–88.8% (20)] occurred during practice, where repetitive skill execution and high training volume increase exposure risk. Structured warm-up protocols, progressive skill development, and enhanced supervision during complex maneuver training are recommended. Education should emphasize spotting mechanics, safe lifting strategies, and early recognition of musculoskeletal and neurological symptoms. Evidence from high school cheerleading suggests that athletes supervised by highly trained coaches experienced nearly 50% fewer injuries compared to those with less educated coaches (28). Early recognition and management of concussion symptoms are particularly important given the contribution of concussions and other head injuries, which accounted for approximately 7% (5, 7–9)–10% (20) of reported injuries. Improving communication between athletes, coaches, and medical personnel helps address reporting barriers, particularly for head injuries. Collegiate athletes were more likely to report injuries when coaches educated them about concussion symptoms (29), enabling earlier care and reducing the risk of long-term consequences. Collectively, these findings support the integration of structured coaching education, medical oversight, and athlete awareness initiatives as core elements of injury prevention strategy in collegiate cheerleading.

Finally, review of the included studies against IOC surveillance recommendations (24) reveals persistent structural gaps in reporting quality. Injury definitions, anatomical classification systems, mechanism coding approaches, and exposure context reporting were inconsistent across studies. Exposure classification was particularly narrow, often restricted to practice or competition settings (19, 20), despite the multidimensional structure of cheerleading participation, which may include pep rallies, cheer competitions, and performances supporting other sports programs. These limitations restrict the development of predictive risk models and reduce the translational utility of existing surveillance data for intervention design.

### 4.1 Limitations of included studies

The studies included in this systematic review were characterized by several methodological limitations. All studies were conducted in the United States and focused primarily on female collegiate athletes participating in NCAA programs, limiting generalizability to other populations, competitive levels, or male athletes (5, 7–9, 19, 20). Study designs were predominantly observational, with several studies relying on voluntary or convenience-based institutional participation. Such recruitment strategies may introduce selection bias, as participating programs may differ in medical oversight, training structure, and surveillance capacity compared with non-participating institutions.

Most studies relied on self-reported injury and exposure data entered by team personnel, introducing potential reporting bias despite structured surveillance procedures in some cohorts (5, 7–9). Injury definition heterogeneity further limited comparability, with some studies capturing only catastrophic injuries (19) while others included injuries requiring time loss and medical attention (5, 7–9, 20). Denominators used for incidence estimation were inconsistent across studies, including athlete-exposures (5, 7–9), total participation counts (19), or unspecified exposure metrics (20). Risk factor modelling and reinjury surveillance were not conducted, and no study systematically evaluated preventive intervention effectiveness. Overall, these limitations suggest that the current epidemiological understanding of collegiate cheerleading injury risk remains incomplete and is derived primarily from descriptive surveillance evidence.

### 4.2 Limitations of this review

This review is limited by the small number of eligible studies and overlapping cohorts, restricting the diversity of data. All included studies were U.S.-based, limiting applicability to international populations. The restriction to English-language publications may have excluded relevant research from non-English-speaking countries, particularly given the growth of cheerleading internationally and the potential for relevant epidemiological data to exist in other languages. Additionally, inconsistencies in study design, injury definitions, and reporting methods hinder the ability to perform quantitative synthesis or meta-analysis. The presence of four studies derived from the same underlying surveillance cohort represents a further limitation, as consistency in findings across these studies reflects shared data rather than independent replication, which constrains the strength of conclusions that can be drawn from the available evidence. Despite these limitations, this review highlights critical gaps in current knowledge and identifies priorities for future research, including the need to adhere to IOC surveillance standards.

### 4.3 Implications

This systematic review underscores the need for improved injury surveillance and prevention infrastructure in collegiate cheerleading. Adherence to IOC consensus guidelines is essential, including standardized injury definitions, detailed anatomical and mechanism coding, and contextual exposure tracking across all participation contexts.

Prospective surveillance systems that incorporate stunts, pyramids, basket tosses, tumbling maneuvers, and fall-related events should be prioritized. Injury categories should include sprains, strains, concussions, fractures, dislocations, and catastrophic head and neck injuries. Such standardized reporting would support identification of modifiable risk factors and facilitate longitudinal outcome evaluation.

All collegiate cheerleading programs should have access to certified ATs, given the current low program coverage (7). Athletic trainers support return-to-play decision-making, concussion management, and injury documentation quality. Injury prevention programs emphasizing lower extremity strength, neuromuscular control, and balance training should be widely implemented, particularly given the high prevalence of ankle injuries observed in surveillance studies (20).

Coaching education programs remain a critical component of injury risk mitigation. Instruction should focus on safe stunt progression, spotting mechanics, and early recognition of musculoskeletal and neurological injury symptoms. Communication pathways between athletes, coaches, and medical personnel should also be strengthened to promote timely reporting and clinical evaluation.

Rule and policy interventions, such as the 2006 ban on basket tosses performed on hard surfaces (19), demonstrate the potential impact of targeted regulatory action. Ongoing evaluation and refinement of safety regulations are necessary to ensure alignment with the evolving technical demands of collegiate cheerleading. Implementation effectiveness of these strategies in collegiate cheerleading populations warrants future empirical evaluation.

### 4.4 Conclusion

Collegiate cheerleading is associated with measurable injury risk, with reported incidence rates in participating programs ranging from 2.4 to 2.8 per 1,000 athlete-exposures, and injuries occurring most frequently during stunts and practice sessions (5, 7–9, 20). Sprains, strains, and concussions represent the most commonly reported injury types. However, the current evidence base is limited in scope, derived from a small-number of U.S.-based studies, several of which rely on overlapping datasets and voluntary institutional participation. Variability in injury definitions, exposure classification, and mechanism reporting further restricts comparability across studies. As a result, the epidemiology of collegiate cheerleading injuries remains incompletely characterized, underscoring the need for contemporary prospective surveillance and standardized reporting practices.

## Data Availability

The original contributions presented in the study are included in the article; further inquiries can be directed to the corresponding author.
